# Rapid Green Synthesis of Silver Nanoparticles by Reishi and Their Antibacterial Activity and Mechanisms

**DOI:** 10.4236/jbnb.2024.153004

**Published:** 2024-07-12

**Authors:** Akamu J. Ewunkem, T’nasia Priester, Desia Williams, Bailey Ariyon, Ilunga Tshimanga, Brittany Justice, Dinesh K. Singh

**Affiliations:** Department of Biological Sciences, Winston-Salem State University, Winston-Salem, North Carolina, USA

**Keywords:** Nanotechnology, Reishi, Nanoparticles, Antimicrobial, Green Synthesis

## Abstract

Nanotechnology is a rapidly growing field in biomedical engineering with references to efficiency, safety, and cost-effective approaches. Herein, the objective of this study was to examine an innovative approach to rapidly synthesis silver nanoparticles from an aqueous extract of medicinal mushroom *Ganoderma lucidum* (also known as reishi). The structural and dimensional dispersion of the biosynthesized silver nanoparticles from reishi was confirmed by UV-Vis spectrophotometer (UV-Vis) and Scanning Electron Microscopy (SEM) analysis. Additionally, the biosynthesized silver nanoparticles from resihi were used to explore their potential antimicrobial activity against *Staphylococcus aureus* and *Micrococcus luteus* and *Escherichia coli* and *Klebsiella pneumoniae*. The results from this study revealed that the silver nanoparticles mediated by reishi adopted a spherical shape morphology with sizes, less than 100 nm and revealed strong absorption plasmon band at 440 nm. Furthermore, the biosynthesized silver nanoparticles from reishi exhibited antibacterial activity against the tested *S. aureus* and *M. luteus* and *E. coli* and *K. pneumoniae* by altering their morphology which signifies their biomedical potential.

## 1. Introduction

The Nobel laureate Richard P. Feynman is often credited with kick-starting nanotechnology back during his famous 1959 lecture “There’s Plenty of Room at the Bottom” meeting of the American Physical Society at the California Institute of Technology (Caltech) [1]. Since then, nanotechnology has rapidly grown and is the most advanced technology innovation or the most promising technology of the 21st century. Nanotechnology provides various important useful applications in a wide range of disciplines from industries, buildings, military, and agriculture to the medical sector [2].

A fundamental area of research in nanotechnology mainly involves the synthesis of nanoparticles of different chemical compositions and dimensions. At present, an increasing number of research is devoted to the synthesis of nanoparticles by chemical, and green synthesis methods [3]. Chemical synthesis of nanoparticles is very expensive and requires toxic chemicals whereas green synthesis is simple, safe, cost-effective, and environmentally friendly. Additionally, green synthesis can produce nanoparticles on a massive scale [4].

Green synthesis of metallic nanoparticles utilizes a wide range of platforms such as plant extracts, bacteria, algae, and fungi. Fungi are attractive agents for green synthesis of metallic nanoparticles due to their high tolerance to metals and are manageability [5]. Fungi have innumerable merits over other organisms since they produce copious extracellular substances that contribute to the stability of the nanoparticles [6].

Mushrooms are macro fungi, which have been used as medicinal products for millennia in many countries. *Ganoderma lucidum* commonly called reishi (in Japan) or lingzhi (in China) is a species of mushroom which has been used for its medicinal properties for a very long time due to the presence of several bioactive compounds [7]. These compounds have anti-inflammatory, radical oxygen scavenging, anti-tumor, immune-enhancing, and antimicrobial activities [8]. The present study explored the synthesis potential of the wild mushroom species *G. lucidum* for manufacturing silver nanoparticles. The antimicrobial potential of the biosynthesized silver nanoparticles from *G. lucidum* was evaluated against selected bacterial strains which included *Staphylococcus aureus* and *Micrococcus luteus* and *Escherichia coli* and *Klebsiella pneumoniae*.

## 2. Materials and Methods

### Collection of Mushroom and Identification

The fresh fruiting bodies of *Ganoderma lucidum* mushrooms named reishi were collected from natural growth in Winston-Salem, North Carolina, USA. The identification of *G. lucidum* was performed by the authors according to the methods of classical herbarium taxonomy; the micro- and macro morphology of collected specimens were compared to standard descriptions in the taxonomic monographs.

### High Performance Liquid Chromatography (HPLC) System

HPLC was carried out in the Department of Chemistry, Winston-Salem State University using Agilent 1100 series with a C18 column (4.6 mm × 250 mm i.d., 5 μm) at 35°C to identify and quantify the bioactive compound in reishi mushroom. The mobile phase consisted of HPLC water (A) and acetonitrile (B) at a flow rate of 1 ml/min. The multi-wavelength detector was monitored at 280 nm.

### Biosynthesis of silver nanoparticles

The synthesis of silver nanoparticles was conducted using *G. lucidum* extract adopting a green synthesis route. Briefly, mushroom extract (10 mL) was combined with 1 mM AgNO_3_ in a conical flask and agitated continuously at room temperature till the appearance of a reddish-brown color.

### Characterization of silver nanoparticles

The silver nanoparticles were characterized by UV-Vis spectrophotometer (UV-Vis) and Scanning Electron Microscope (SEM). The biosynthesized silver nanoparticles were monitored and measured using GENESYS^™^ 180 UV-Vis Spectrophotometer (Fisher Scientific, USA) within the range of 200 - 1000 nm. Furthermore, morphology and size of the biosynthesized nanoparticles was observed by SEM (JEOL JSM-IT800 HL, JEOL Ltd, Japan) at the Joint School of Nanoscience and Nanoengineering, University of North Carolina at Greensboro and North Carolina A and T State University, Greensboro, North Carolina, USA. Briefly, a freeze-dried sample of silver nanoparticle solution was sonicated with distilled water. Afterward, a small drop of the sample was placed on glass slide and allowed to dry. A thin layer of platinum was coated to make the samples conductive. The Jeol JSM-6480 LV SEM machine was operated at a vacuum of the order of 10 - 5 torr. The accelerating voltage of the microscope was kept in the range 10 - 20 kV.

### Antimicrobial activity of biosynthesized silver nanoparticles from reishi

Promising antimicrobial activity of the biosynthesized silver nanoparticles from *G. lucidum* was tested against *Escherichia coli* 1946 (ATCC 25922), *Staphylococcus aureus* (ATCC 25923), *Klebsiella pneumoniae* NCTC 9633 (ATCC 13883) and *Micrococcus luteus* (ATCC 4698) using broth dilution method in the 96-well plates. The bacterial cultures were treated with various concentrations (0 - 30 μM) of the biosynthesized silver nanoparticles. The plates were incubated for 24 h at 37°C with agitation at 120 rpm in a shaking incubator. Growth of the tested bacteria was measured at 24 hr using a 98-well plate format Glomaxmulti plate reader (Promega, USA).

### Statistical Analysis

All analyses were carried out in triplicates and the data are expressed as mean ± standard deviation (SD). GraphPad Prism software was used for statistical analyses. Statistical analysis was performed using the 2-tailed, independent t-test. Statistical significance was set at P < 0.05.

## 3. Results

### HPLC Analysis

**Figure 1.**
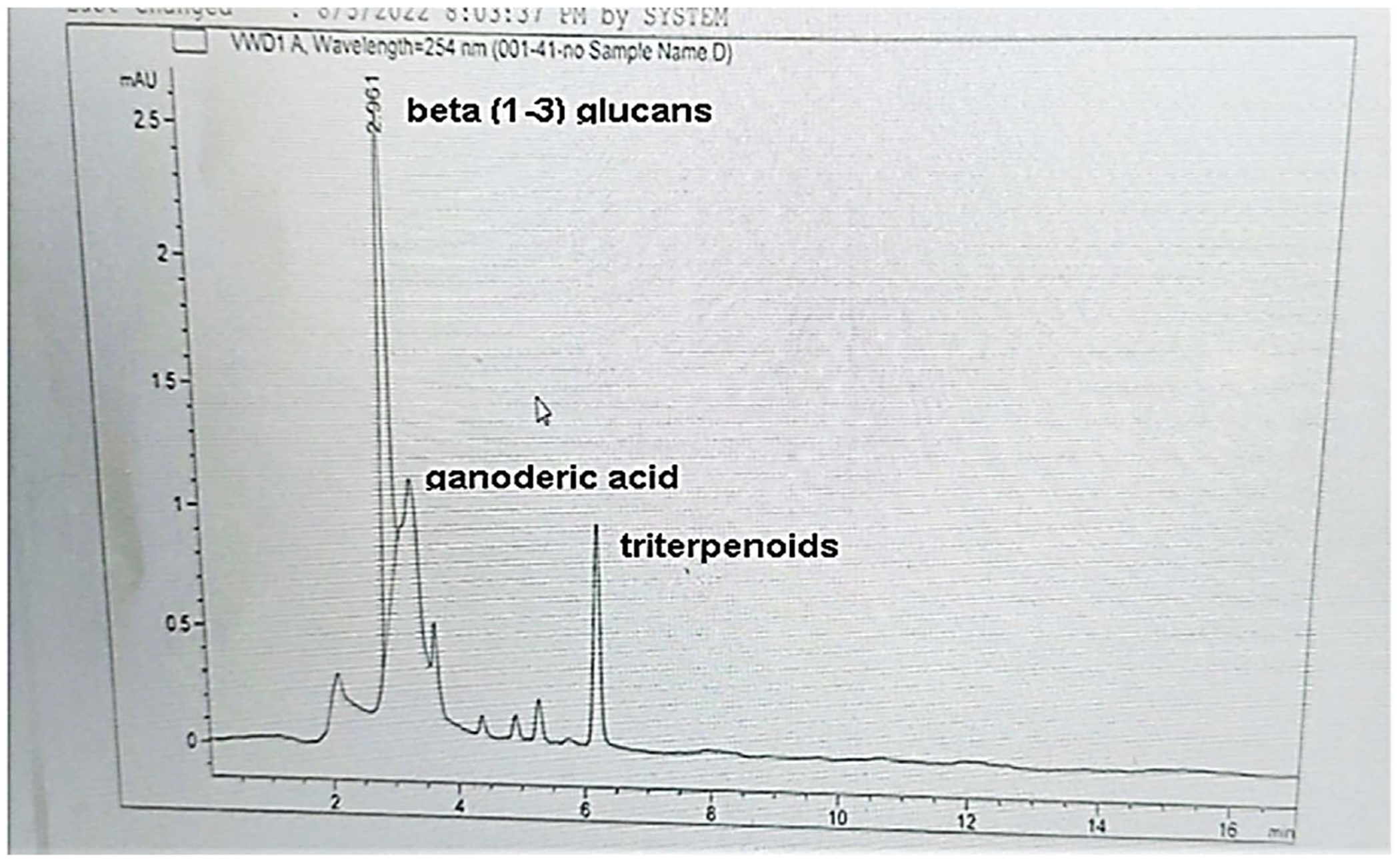
HPLC profile of aqueous extract derived from reishi.

The content of the polyphenolic compounds in aqueous extract of reishi and retention times determined by HPLC analysis are shown in Figure 1. HPLC revealed three main spikes representing beta (1 - 3) glucans, ganoderic acid and triterpenoids.

### Biosynthesis and Characterization of Silver Nanoparticles

**Figure 2.**
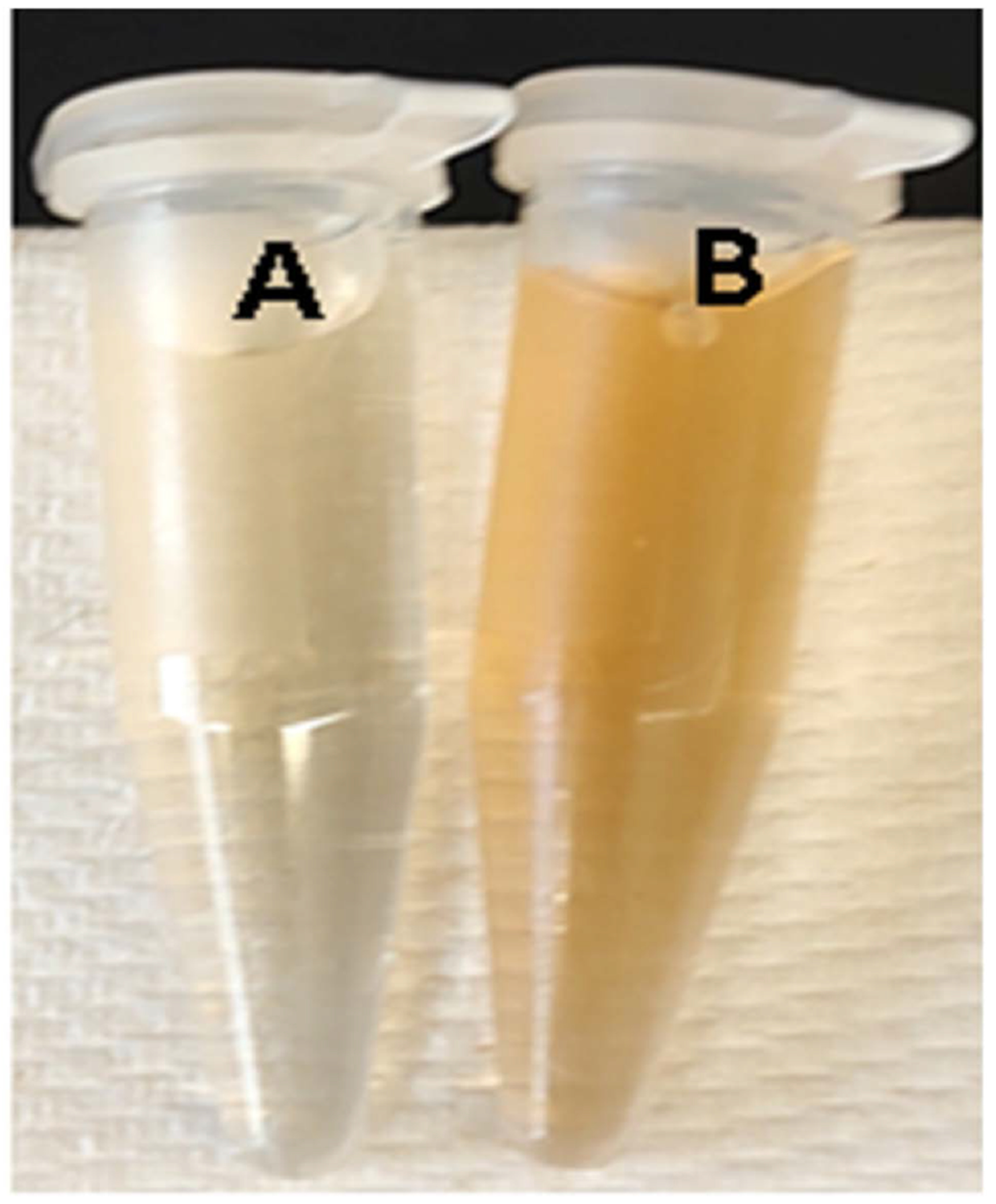
Color change observed in reishi extract after exposure to AgNO_3_ at 28°C with agitation. A reishi extract and AgNO_3_; (B) synthesized silver nanoparticles.

Synthesis of silver nanoparticles using reishi extract was observed (Figure 2). When the reishi extract was subjected to aqueous solution, a gradual change of the pale yellow-colored (AgNO_3_ + reishi extract) (Figure 2(A)) after 1 hour to yellowish brown (Figure 2(B)) confirmed the synthesis of silver nanoparticles. The yellowish brown remained unchanged even after 168 hours suggesting that the biosynthesized silver nanoparticles were dispersed in the solution.

**Figure 3.**
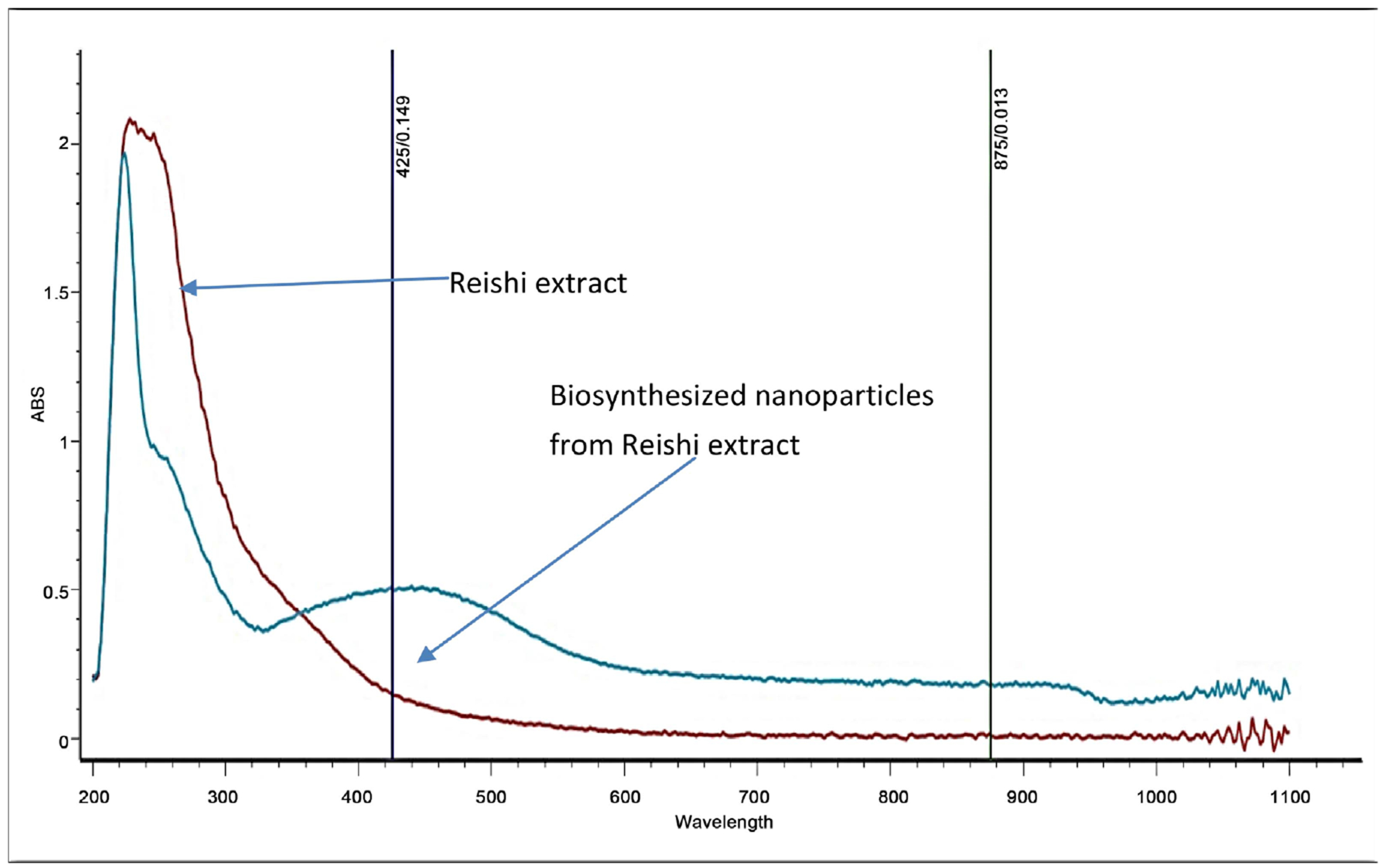
The ultraviolet-visible spectra of biosynthesized silver nanoparticles from reishi.

**Figure 4.**
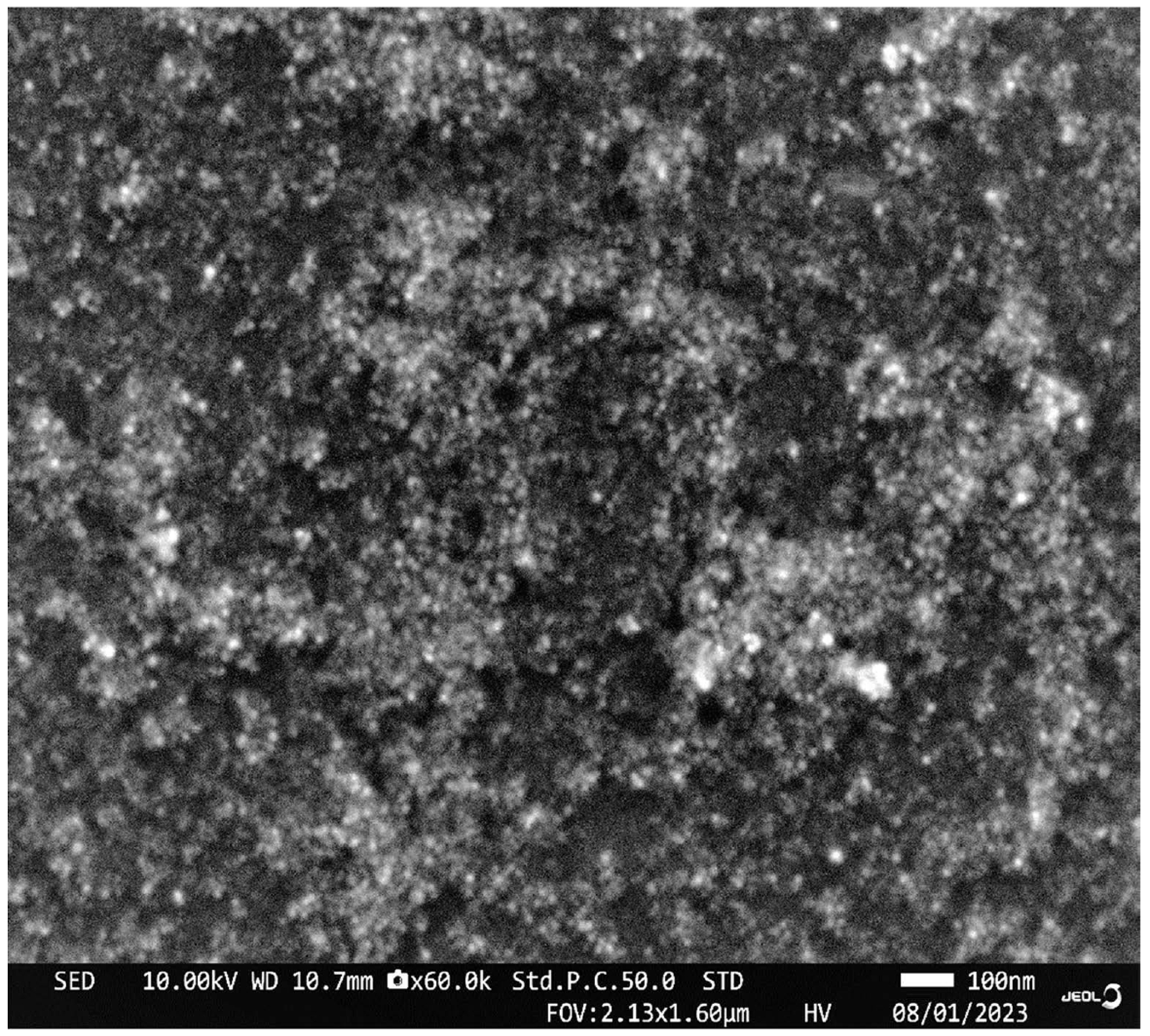
Representative scanning electron microscopy micrograph of biosynthesized silver nanoparticles derived from reishi.

The silver nanoparticles obtained by reishi mushroom mediated green synthesis method, were characterized by Ultra-violet visible (UV-vis) spectroscopy and Scanning Electron Microscope (SEM). UV-Vis spectrophotometry is a decisive and reproducible technique that is suitable for the accurate characterization of metal nanoparticles. The biosynthesized silver nanoparticles showed higher absorption peak (440 nm) compared to the reishi extract (Figure 3). The higher absorption peak corresponded to surface plasmon resonance (SPR) of the silver nanoparticles. SEM provides images for visualizing the shape, size and aggregation of nanoparticles. SEM revealed the occurrence of the biosynthesized silver nanoparticles from reish in a spherical shape with, less than 100 nm in size (Figure 4).

### Antimicrobial activity of biosynthesized silver nanoparticles from reishi

**Figure 5.**
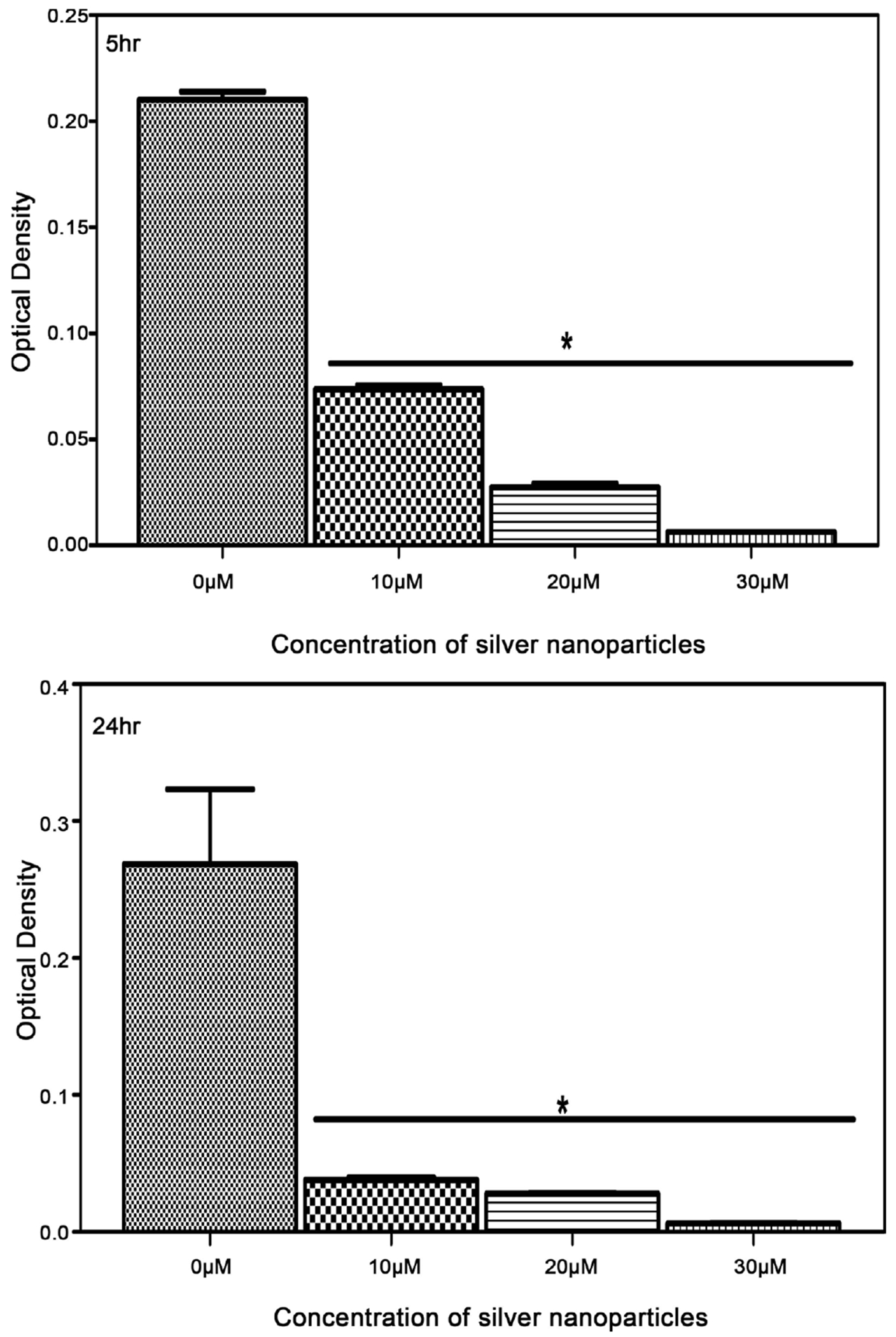
Impact of biosynthesized silver nanoparticles from reishi on the growth of *Staphylococcus aureus* (Optical density Measurement) for 5 and 24 hr.

**Figure 6.**
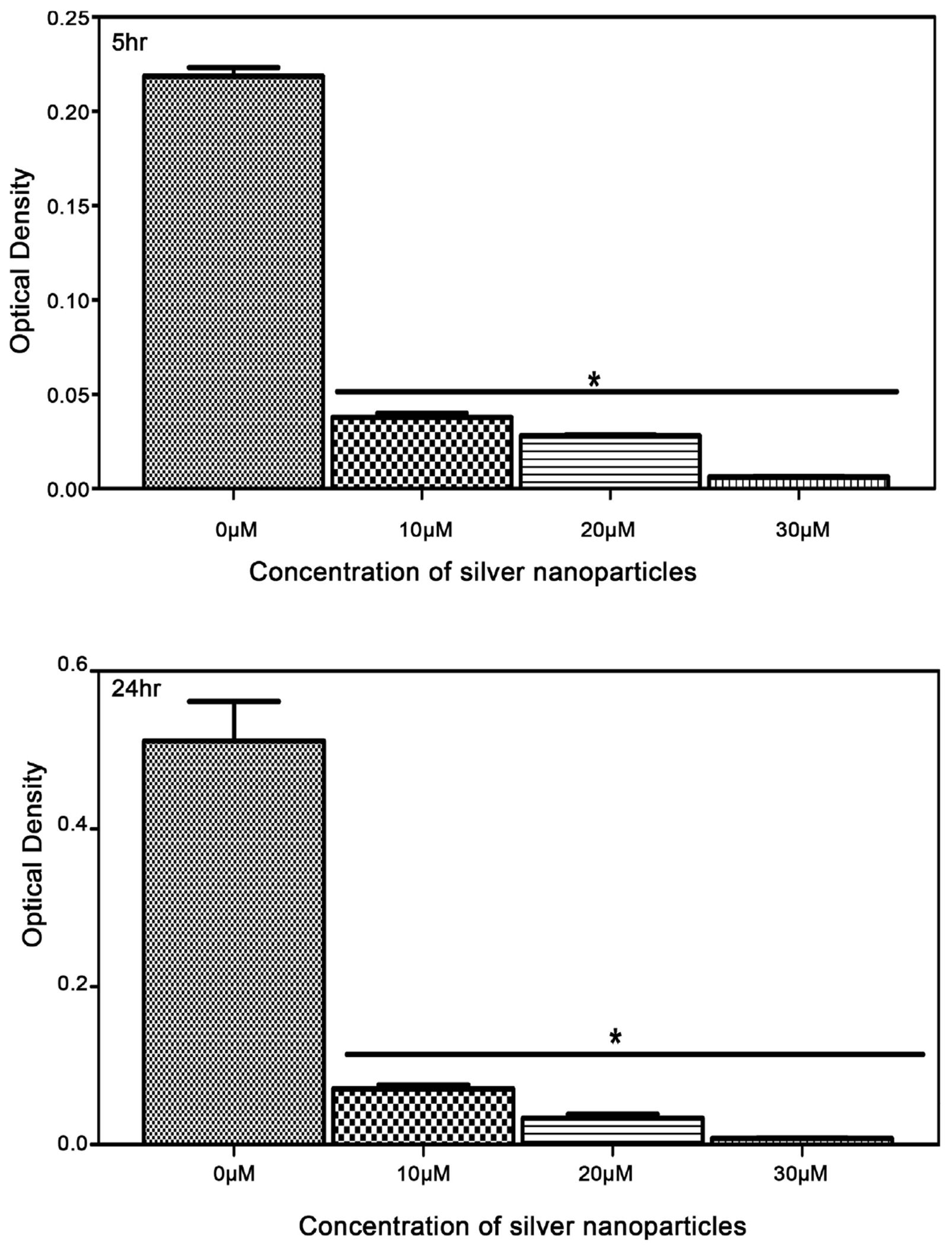
Impact of biosynthesized silver nanoparticles from reishi on the growth of *Micrococcus luteus* (Optical density *Measurement*) for 5 and 24 hr.

**Figure 7.**
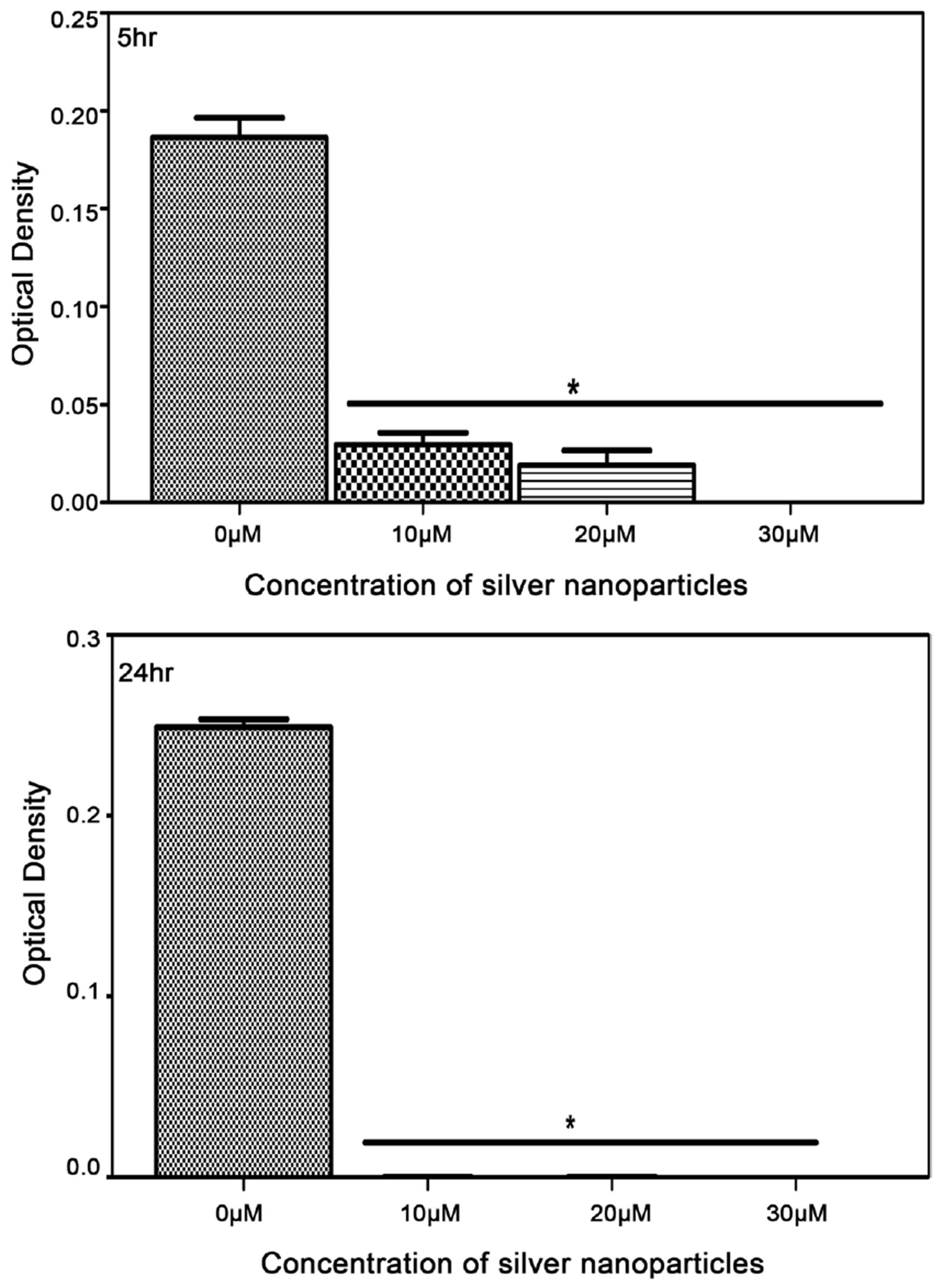
Impact of biosynthesized silver nanoparticles from reishi on the growth of *Klebsiella pneumoniae* (Optical density Measurement) for 5 and 24 hr.

**Figure 8.**
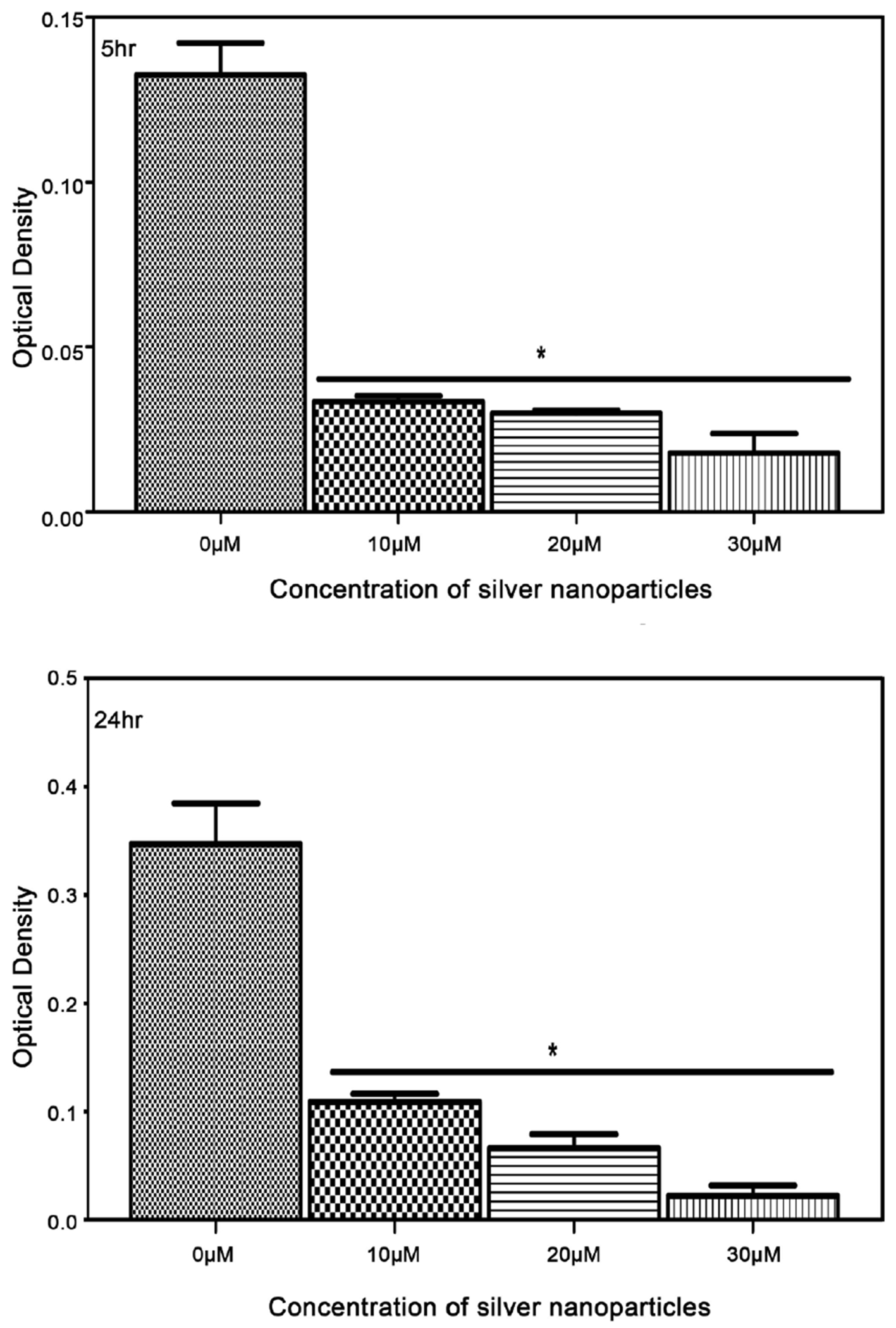
Impact of biosynthesized silver nanoparticles from reishi on the growth of *Escherichia coli* (Optical density Measurement) for 5 and 24 hr.

In vitro antimicrobial of biosynthesized silver nanoparticles from reishi was assessed by broth dilution against *M. luteus, K. pneumoniae, E. coli*, and *S. aureus*. The results showed positive inhibitory activity of silver nanoparticles against all tested bacterial strains (Figures 5–8). For the control bacteria, an increase in optical density was observed over time indicating bacterial growth. The biosynthesized silver nanoparticles from reishi significantly reduced the bacterial growth at all time points. Bacterial responses to the biosynthesized silver nanoparticles were concentrated-dependent and time-dependent. At higher concentrations, and 24 h post exposure, the silver nanoparticles exhibited a stronger antimicrobial activities on *M. luteus, K. pneumoniae, E. coli*, and *S. aureus*.

The antibacterial activity of the biosynthesized silver nanoparticles against Gram-positive bacteria (Figure 5 and Figure 6) showed that all biosynthesized silver nanoparticles had efficient antibacterial activity against *S. aureus* and *M. Luteus* after 5 and 24 hr of incubation. Biosynthesized silver nanoparticle induced at least 50% reduction in growth of *S. aureus* and *M. luteus*. *M. luteus* proved to be more sensitive to the presence of silver nanoparticles (Figure 6).

The growth inhibition was most noticeable for Gram negative bacteria with increasing antibacterial activity observed at higher concentrations of the biosynthesized silver nanoparticles and at longer time points (Figures 6–8). At 5 hr and 24 hr post exposure, the biosynthesized silver nanoparticles caused at least 65% reduction in both *K. pneumoniae* and *E. coli* (Figure 7 and Figure 8) when compared to the control (untreated cells). Strongest growth inhibition was observed after 24 hr.

**Figure 9.**
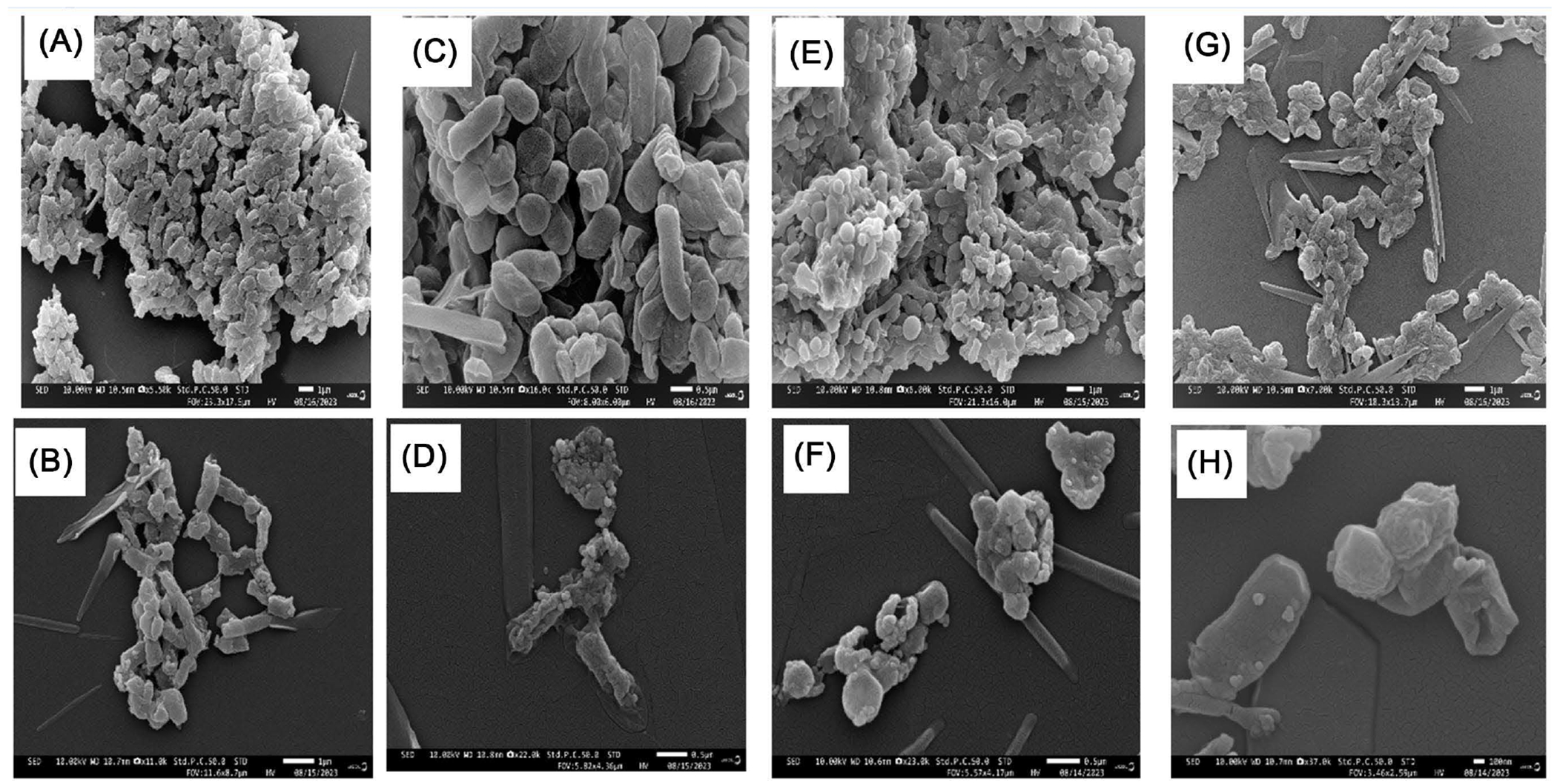
SEM images of control bacterial cells and cells treated with biosynthesized nanoparticles from reishi after 24 h. (A) Control *E. coli*; (B) *E. coli* treated with silver nanoparticles; (C) Control *K. pneumonia*; (D) *K. pneumonia* treated with nanoparticles; (E) Control *S. aureus*; (F) *S. aureus* treated with nanoparticles; (G) Control *M. luteus*; (H) *M. luteus* treated with silver nanoparticles.

Morphological and ultrastructural changes of *S. aureus, M. luteus, E. coli* and *K. pneumonia* cells treated with biosynthesized silver nanoparticles were directly observed by SEM (Figure 9). By examining the SEM, the untreated cells were seen normal as typical spherical or cocci-shaped (*S. aureus*) or rod-shaped (*E. coli, M. luteus*, and *K. pneumonia*) with no damage to cell surfaces (Figure 9(A), Figure 9(C), Figure 9(E) and Figure 9(G)). However, treated cells with 30 μM of the biosynthesized silver nanoparticles attached were seen attached to the bacterial cell wall (Figure 9(B), Figure 9(D), Figure 9(F) and Figure 9(H)). The normal shape of cells was changed and appeared as distorted and damaged.

## 4. Discussion

Nanotechnology is a promising technology and has attracted scientists for more than twenty years. An important field of research in nanotechnology is the synthesis of nanoparticles. The present study highlights the rapid synthesis of silver nanoparticles using reishi mushroom *Ganoderma lucidum* and their antimicrobial mechanisms against *S. aureus, M. luteus, K. pneumonia* and *E. coli*. The biosynthesized silver nanoparticles method in this study was reliable, straightforward, and rapid.

During the biosynthesis of silver nanoparticles, a visible color change of the mixture AgNO_3_ solution and reishi extract was observed within an hour of incubation with the reishi extract turning to pale brown confirming the rapid synthesis of silver nanoparticles. The color change is due to the change in surface plasmon resonance of silver nanoparticles and the reduction of AgNO_3_ [9] [10] [11]. Additionally, the presence of bioactive compounds in reishi (Figure 1) may have contributed to the recovery of silver ions and the formation of silver nanoparticles as suggested by previous studies [12] [13].

Silver nanoparticles are known to absorb light and generally have peaks near 400 nm and larger sphere display increased scattering and produce peaks that broaden and shift towards longer wavelengths (14). This can also be confirmed by taking absorbance using UV-Vis spectroscopy because silver nanoparticles exhibit their maximum absorbance in the UV range [14]. The synthesis of silver nanoparticles from reishi in the flask was further confirmed with the UV-Vis characteristic absorption band of silver nanoparticles at a wavelength of 440 nm providing again that the successful reduction of silver ion and the formation of silver nanoparticles. It has been reported that the silver nanoparticles exhibit a UV-visible absorption maximum in the range of 410 - 450 nm [11] [15] [16].

The morphology and size distribution of nanoparticles are indispensable for understanding their behavior and performance. Scanning electron microscope (SEM) is one of the most highly effective imaging techniques used to examine the morphology and size of nanoparticles [17]. SEM results revealed that the biosynthesized silver nanoparticles from reishi were spherical shaped and had diameter ranging less than 100 nm which are in accordance with the previous results [11] [18] [19].

Nanoparticles morphology is an important factor underpinning its effectiveness in therapeutic applications [20]. Nanoparticles have increasingly been used to target pathogens [11] [19] [21]. The antibacterial activity of biosynthesized silver nanoparticles from reishi was studied against pathogenic bacterial strains of gram-negative (*E. coli* and *K. pneumonia*) and gram-positive (S. *aureus* and *M. luteus*). The biosynthesized silver nanoparticles have strong antibacterial activity against both gram-negative and gram-positive bacteria by damaging the cell wall and membrane of the bacterial cells resulting in various morphological changes as seen in other studies [11] [19]. The silver nanoparticles are not the main antimicrobial agents. The silver nanoparticles are seen as silver ion reservoir and when attached to bacterial cell walls they release silver ions which then interact with the cell membrane and disrupt cellular activities leading to bacterial death [22] [23] [24]. The presence of bioactive components in the reishi extracts might have enhanced the antimicrobial activity of the biosynthesized silver nanoparticles [24]. The Gram-negative bacteria were more susceptible to the biosynthesized silver nanoparticles than Gram-negative bacteria due to differences in the nature of the cell wall [25]. Gram-negative bacteria have thin peptidoglycan layer in their cell wall with thick lipopolysaccharide layer. In contrast Gram-positive bacteria have a thick peptidoglycan layer and no outer lipid membrane [26]. The thick peptidoglycan in gram positive bacteria makes it harder for the silver nanoparticles to penetrate the cell wall [27].

## 5. Conclusion

Reishi mushrooms (*Ganoderma lucidum*) are widely used as traditional medicines to boost immune system and promote health. In this report, we used reishi to rapidly synthesize silver nanoparticles. The biosynthesized silver nanoparticles exhibited potential antibacterial activity against Gram-negative and Gram-positive bacteria. The data from this study suggest that biosynthesized silver nanoparticles can be used for pharmacological and medicinal purposes. This approach proves to be rapid and green for the synthesis of silver nanoparticles.
